# Electrophysiological evidence for an early processing of human voices

**DOI:** 10.1186/1471-2202-10-127

**Published:** 2009-10-20

**Authors:** Ian Charest, Cyril R Pernet, Guillaume A Rousselet, Ileana Quiñones, Marianne Latinus, Sarah Fillion-Bilodeau, Jean-Pierre Chartrand, Pascal Belin

**Affiliations:** 1 Centre for Cognitive NeuroImaging (CCNi) & Department of Psychology, University of Glasgow, Glasgow, UK; 2 SFC Brain Imaging Research Centre, Division of Clinical Neurosciences, University of Edinburgh, Edinburgh, UK; 3 Cuban Neuroscience Centre, Department of Cognitive Neuroscience, Havana, Cuba; 4 Département de Psychologie, Université de Montréal, Montréal, QC, Canada; 5 International Laboratory for Brain, Music and Sound Research, Université de Montréal & McGill University, Montreal, Canada

## Abstract

**Background:**

Previous electrophysiological studies have identified a "voice specific response" (VSR) peaking around 320 ms after stimulus onset, a latency markedly longer than the 70 ms needed to discriminate living from non-living sound sources and the 150 ms to 200 ms needed for the processing of voice paralinguistic qualities. In the present study, we investigated whether an early electrophysiological difference between voice and non-voice stimuli could be observed.

**Results:**

ERPs were recorded from 32 healthy volunteers who listened to 200 ms long stimuli from three sound categories - voices, bird songs and environmental sounds - whilst performing a pure-tone detection task. ERP analyses revealed voice/non-voice amplitude differences emerging as early as 164 ms post stimulus onset and peaking around 200 ms on fronto-temporal (positivity) and occipital (negativity) electrodes.

**Conclusion:**

Our electrophysiological results suggest a rapid brain discrimination of sounds of voice, termed the "fronto-temporal positivity to voices" (FTPV), at latencies comparable to the well-known face-preferential N170.

## Background

The field of study of cortical processing of complex sounds has been highly productive in the recent past both in humans and monkeys. A model similar to the "what" and "where" segregation of the visual processing network has been suggested for auditory processes of sound identification and localization [1,2]. Regions within the superior and middle temporal cortices and the inferior prefrontal gyrus have been identified as candidates for the "what" pathway of the auditory stream, whereas a "where" pathway would rely on the posterior temporal cortex and the inferior and superior parietal cortices [3-6]. Within the auditory "what" pathway, functional magnetic resonance imaging (fMRI) studies have identified the 'temporal voice areas' (TVA - [7]), i.e. bilateral auditory areas situated along the superior temporal sulcus (STS) showing a greater response to human vocalisations than to other sound categories [8,9]. These regions were later found to be species-specific as they elicited stronger responses to human vocalisations compared to non-human vocalisations [10,11]. In a recent fMRI study in macaques, Petkov et al. (2008) found a region of the secondary auditory cortex which showed a comparable preference for conspecific vocalisations over vocalisations from other species or non-vocal sounds. These findings suggest a long evolutionary history of voice-preferential processing [12]. Yet, the time course of voice processing remains unclear.

In studies using intracranial electrophysiological recordings in human participants, early responses to sound stimulation were shown to reach the primary auditory cortex (A1) as early as 15 ms after sound onset [13-15] and differences between sound categories have been observed as soon as 55 ms after this early response to sound stimulation. Using evoked related potentials (ERPs) and an oddball paradigm, Murray et al. (2006) reported early ERP differences between man-made (sound of a bicycle bell, glass shattering, telephone...) and living auditory objects (baby cries, coughing, birdsong, cat vocalization...) as early as 70 ms after stimulus onset [16]. Although most of the living sounds in that study consisted of vocalisations, it remains unclear whether the effect was driven by voices or not. ERP studies providing evidence directly relevant to the speed of voice/non-voice categorisation are scarce. Two studies found a larger response to sung voices when compared to instrumental sounds at a latency of 320 ms after stimulus onset, with a fronto-central distribution, which was termed the "voice-specific response" (VSR) [17,18]. To further assess the VSR, Gunji et al. (2003) used magnetoencephalography (MEG) and analysed two components of the evoked response: the N1m and the sustained field observed 400 ms after stimulus onset. They observed no difference in the magnitude of the N1m component between voices and instrumental sounds; however the source strength of the 400 ms sustained field was larger for vocal sound than for instrumental sounds [19]. Both components had sources in Heschl's gyrus in both hemispheres.

Although previous studies did not address directly the voice/non-voice discrimination process, some of them suggest the existence of earlier correlates of voice processing. Indeed, effects of voice familiarity [20], voice gender adaptation [21], human vs. computer voice [22], voice priming [23], speech vs. tones [24], and speaker identity [25] have been observed between 150 ms to 200 ms.

The relatively long latency of voice vs. non-voice ERP differences (320 ms) stands in strong contrast with these results and with the early living/non-living distinction reported by Murray et al. (2006).

In the present study, we investigated the speed of voice processing by measuring ERPs in response to sounds from three categories -- voices, bird songs and environmental sounds -- while participants were performing an incidental target (pure tone) detection task. We hypothesised that since neural correlates of voice paralinguistic characteristics were observed in the range of 150 to 200 ms, investigating neural correlates of voice recognition by directly comparing neural responses to voices with those of sounds from other categories should lead to differences in the same latency range or earlier, in contrast with the previously reported 320 ms.

## Methods

### Participants

Thirty-two French-speaking adults (15 females, mean = 27.25 y/o, std. 8.23), participated in the study. They all reported normal audition and no neurological problems. They all gave informed written consent and the study was approved by the University of Montreal ethics committee. Participants were compensated 30 Canadian dollars for their time. Some of the subjects were included in the 'novice' group of a study focusing on differences between ornithologists and novices [26], although they were never informed of the expertise nature of this study, and only informed to press a response button as fast as they could when they heard a 1000 Hz pure tone target.

### Stimuli and design

Stimuli consisted of 450 sound samples, 150 in each of three categories: bird songs, human vocalisations (73 speech items, 77 vocalisations) and environmental sounds (30 natural sounds, 60 instruments and 60 mechanical sounds). The bird songs were selected from the « Chants d'oiseaux du Québec et de l'Amérique du Nord » (2004; Peterson Guides coll, Broquet/Cornell laboratory of ornithology) audio CD. Other sound stimuli came from commercially available sources and from recordings in the laboratory. Sounds were edited using Cool Edit Pro (Syntrillium Corporation, Phoenix, Arizona, USA) to a sampling rate of 22050 Hz, a 16-bit resolution, and duration of 200 ms with a 10-ms linear attack and decay. They were all root mean square (RMS) normalised using Matlab (The MathWorks Inc., Natick, Massachusetts, USA). A sample of the stimuli is available for consultation online .

**Figure 1:**
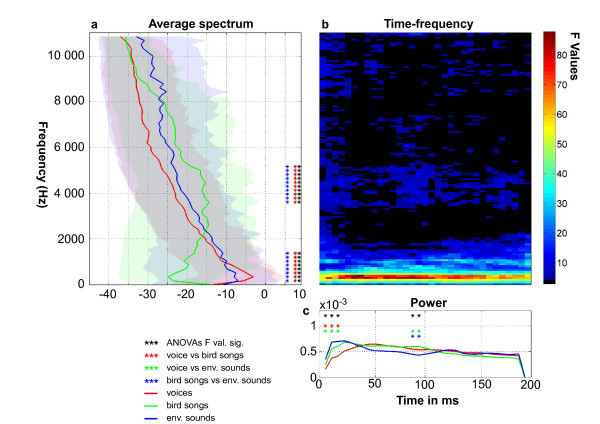
**Acoustical differences between sound categories**. a) Red, blue and green lines show the average power spectrum of their respective sound categories (see legend). Stars highlight frequency bands showing a significant effect of sound category. 95% confidence intervals are indicated by shaded areas. b) Time-frequency analysis. The significance of the sound categorical difference effect is indicated by the colormap. Black areas indicate non-significant results. Frequency axis as in figure 1a. c) Power Analysis in time. Red, blue and green lines show the average power of the respective sound categories over the 200 ms duration. Black stars indicate time points showing a significant effect of sound category; blue and green stars indicate significant post-hoc tests. Note that the largest effect of sound category is a higher power in the low frequencies for voices and environmental sounds compared to bird songs.

Analyses of sound power in the temporal, spectral and time-frequency domains were performed using one-way ANOVAs at each time, frequency, or time-frequency bin (11.6 ms, 43 Hz) using Matlab (figure 1). In the time domain, power differences between the three sound categories were observed from 11 to 35 ms (minimum F = 8.05, p < 0.05) and from 76 to 100 ms (minimum F = 8.08, p < 0.05; figure 1c). Post-hoc tests showed that differences in the temporal domain were driven by voices from 11 to 35 ms and by environmental sounds from 76 to 100 ms, with significantly less power than the other two categories(p < 0.05).

In the frequency domain, significant effects were found around 500 Hz (minimum F = 25.52, p < 0.05) and 4000 Hz (minimum F = 20.09, p < 0.05; figure 1a), reflecting the smaller power in low frequencies (0-2000 Hz) and greater power at intermediate frequencies (2000-8000 Hz) for bird songs relative to the other two categories (p < 0.05); and more power at high frequencies (> 8000 Hz) for environmental sounds compared to the other two categories although this last difference was not significant (p > 0.05).

In the time-frequency domain, significant effects of sound category were observed across all frequencies for early latencies only (0-30 ms; p < 0.05), whilst differences in frequency bands around 500 Hz and 4000 Hz were observed at all time-bins (p < 0.05; figure 1b).

### Procedure: sound identification

10 participants were included in a verification study in which they listened to each sound from the three stimulus categories and performed a three alternative forced choice categorisation task. All categories showed similar levels of recognition (percent correct ± standard deviation: 92 ± 4% for voices, 90 ± 10% for bird songs and 85 ± 8% for environmental sounds). Statistical analyses testing the null H0 hypothesis with a bootstrap method (1000 samples with replacement of the categorical labels; procedure explained in more details in the EEG recordings and analysis section) showed no significant differences between the three categories (p > 0.05).

### Procedure: Task

Participants were seated in a sound-proof cabin and were presented with each sound from the three categories in a pseudo-random order with a 3000-3500 ms random inter-stimulus-interval (ISI). Each sound was played twice, in two different runs. Stimuli were presented via Beyerdynamic DT 770 headphones at a self-adjusted comfortable level of about 65 dB sound level, as measured using a Lutron Sl-4010 sound level meter. Participants were instructed to detect a 1000 Hz sinusoidal pure sound target with a 10% probability of occurrence. They were instructed to press a button each time they heard the target stimulus, and also to minimise blinking, head motion and swallowing.

### Procedure: EEG recordings and analysis

Electroencephalography (EEG) data were recorded continuously at a 250 Hz sampling frequency using a BrainAmp amplifier (Brainproduct-MR 64 channel-Standard; 62 EEG electrodes, one EOG, one ECG, Brain Products, Munich, Germany) using a 0.5-70 Hz band-pass filter. The 64 Ag/AgCl electrodes were attached using a BrainCap 10-20 array, with an on-line reference at electrode FCz, a ground electrode on the midline posterior to Oz, an ECG electrode attached above the left collar bone, and an EOG electrode attached above the zygomatic bone, below the left eye. The electrode impedances were kept below 10 kΩ throughout the recording.

EEG recordings were analysed using EEGLAB v6.01 [27], under Matlab (The MathWorks Inc., Natick, Massachusetts, USA). Trials with abnormal activities were excluded based on a detection of extreme values (± 100 *μ*V for all channels), abnormal trends (trial's slope larger than 75 *μ*V/epoch and a regression *R*^2 ^larger than 0.3), and abnormal distribution (when the trial's kurtosis fell outside five standard deviations of the kurtosis distribution for each single electrode or across all electrodes) [27,28]. Data were then re-referenced to the average of all electrodes and band-pass filtered in the range 1-30 Hz. For each stimulus category, EEG epochs of 1200 ms, starting 200 ms before stimulus onset, were averaged, and the mean pre-stimulus activity was subtracted from the activity at each time point.

Statistical inferences on amplitude differences between sound categories were performed for each electrode and time point using bootstrap procedures implemented in Matlab (The MathWorks Inc., Natick, Massachusetts, USA). The null hypothesis H0 that the three conditions were sampled from populations with similar means was evaluated by sampling with replacement, independently for each subject, among the three categories. The samples consisted of the full electrodes by time-points matrices. This was followed by averaging the ERP across subjects for each resampled condition, and then computing the differences between the means of two fake conditions. This process was repeated 9999 times, leading to a distribution of 10000 bootstrapped estimates of the mean difference across subjects between two ERP conditions. Then the 99.9% percent confidence interval was computed (alpha = 0.001). Finally, the difference between two sample means was considered significant if it was not contained in the 99.9% null hypothesis confidence interval [29,30]. The statistical analyses were restrained to a [-200 to 500 ms] time-window as we were mainly interested in rapid brain discrimination processes.

In order to evaluate the relative contribution of speech and non-speech vocal sounds, post-hoc analyses were performed on a sample of 50 speech sounds (vowel, word, consonant, etc.) and 50 non-speech vocal sounds (cough, laughter, yawn, gargle, etc.) selected from the voice category (the 50 most ambiguous were excluded from the analysis). Electrophysiological responses to these two categories were then compared independently to the average of bird songs and environmental sounds using the bootstrap procedure described above.

## Results

### Behavioral results: target detection task during EEG recordings

Mean reaction times (RT) to correctly detected targets were 563 ms across subjects. In terms of accuracy, 97.82% of the targets were followed by a button press (hits) and only 0.04% of the non-target events were followed by a button press (false alarms (FA)).

Responses to targets were split according to whether targets followed voices, bird songs or environmental sounds. Repeated measures ANOVAs were implemented on RT for correct responses, proportion of hits and false alarms using SPSS (SPSS, Chicago, Illinois). We did not observe differences in proportion of hits (F(2,62) = 0.532, p = 0.590), nor in FA rate (F(2,62) = 1.882, p = 0.161), but we observed a significant difference in RT (F(2,62) = 6.745, p = 0.002). Paired samples t-tests indicated significantly longer reaction times in response to the pure tone following presentation of bird songs than for voices (t = -4.63, df = 31; p < 0.001) and environmental sounds (t = 2.76, df = 31; p < 0.01), which did not differ from each other (t = -0.357, df = 31; p = 0.724). Mean values and their standard deviations for the behavioral results are reported in Table 1.

**Table 1:** Results of the target detection task during ERPs

	***RT (ms)***	***% Hits***	***% Miss***	***% FA***
Voices	553 ± 174	98.12 ± 4.79	1.88 ± 4.79	0.08 ± 0.32

bird songs	581 ± 165	97.71 ± 4.87	2.29 ± 4.87	0.02 ± 0.05

environmental sounds	556 ± 156	97.63 ± 6.17	2.37 ± 6.17	0.14 ± 0.18

Average reaction times (RT, in ms), and proportion of hits and miss were split depending on whether the pure tone target was preceded by the presentation of voices, bird songs or environmental sounds. The table also presents the relevant standard deviations from the mean. Proportion of false alarms following the presentation of the 3 sound categories are also presented in the table with standard deviations from the mean.

### Event Related Potentials

#### Voices vs. bird songs and environmental sounds

**Figure 2:**
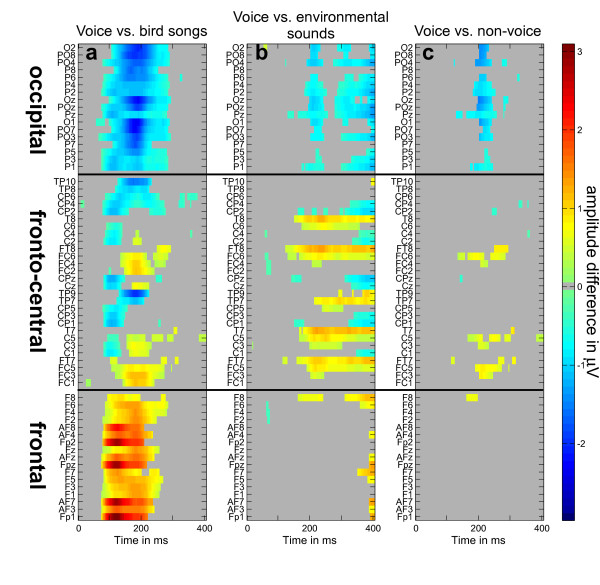
**ERP bootstrap results**. Significant ERP amplitude difference at all electrodes (vertical axis). Gray areas represent non-significant ERP amplitude differences. Significant average ERP amplitude differences (μV) are represented in an increasing gradient from blue to red (jet64). Bootstrap tests revealed significant amplitude differences between (a) voices vs. birdsongs, (b) voices vs. environmental sounds, and (c) voices vs. both birdsongs and environmental sounds (conjunction of (a) and (b)).

ERPs in all participants showed the classical N1-P2 waveform components with central topographic distribution [31-34]. Results from the comparison of ERPs to voice vs. the other categories are shown in figure 2. The earliest amplitude differences were observed for the voice vs. bird song comparison, emerging around 64 ms after sound onset at electrodes O1, PO3 and PO4 (figure 2a), and about 10 ms later at most of the occipital and frontal electrodes. These differences peaked at about 200 ms, and lasted until 300 ms after stimulus onset. The earliest amplitude differences between voices and environmental sounds were observed at 120 ms on fronto-temporal electrodes FT8 and FT7, peaking at about 200 ms and lasting until 400 ms after stimulus onset (figure 2b). Significant amplitude differences between voices and environmental sounds were also observed around 200 ms on several occipital electrodes (figure 2b). A conjunction of these two differences revealed a broadly distributed pattern of ERPs with a preferential response to voices (figure 2c). While bilateral fronto-temporal electrodes (FC5, FC6) showed a greater positivity in response to voices, a larger negativity was observed at occipital locations (PO7, PO8).

**Figure 3:**
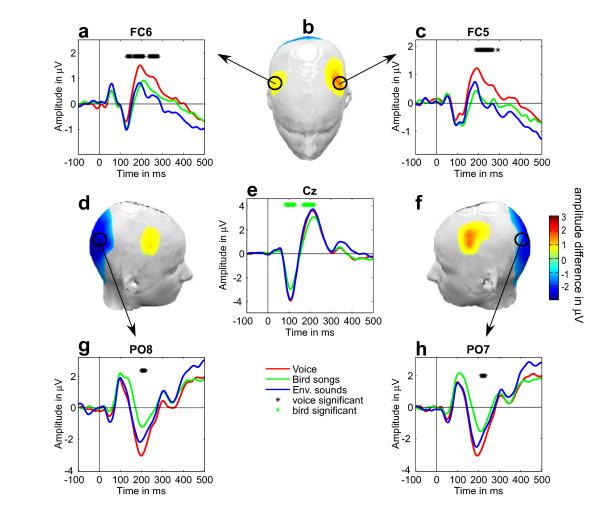
**Electrophysiological responses and bootstrap results**. a, c, e, g, h) Colored lines indicate ERP waveforms for each category. Black stars indicate 4 ms time bins at which electrophysiological responses to voices was significantly stronger than both the two other stimulus categories (voice > non-voice). Green circles indicate 4 ms time-bins at which ERPs bird songs were significantly greater than to both voice and environmental sounds. b, d, f) Headplot showing the voice > non-voice significant difference at 200 ms and electrode positions for panels a, c, g and h. Colormap indicates significant amplitude difference (in μV) with greater ERP amplitude (positive or negative) for voices compared to both bird songs and environmental sounds. Gray areas were not significant.

At fronto-temporal electrode FC6 (right hemisphere), a significant amplitude difference showing a smaller negative potential for voice compared to both bird songs and environmental sounds was observed between 132 ms and 152 ms (figure 3a). Following this smaller negativity, a larger positive ERP amplitude elicited by voice sounds compared to bird and environmental sounds was observed at fronto-temporal electrode FC6 as early as 164 ms extending to 280 ms (figure 3a), and from 188 ms to 268 ms at fronto-temporal electrode FC5 (left hemisphere; figure 3c). At occipital electrodes, larger ERP negativities for voices compared to both bird songs and environmental sounds were observed from 200 ms to 220 ms with maximal difference at 200 ms at electrode PO8 (right hemisphere, figure 3g), and from 212 ms to 232 ms with maximal difference at 212 ms at electrode PO7 (left hemisphere, figure 3h).

#### Speech contribution to the voice effect

**Figure 4:**
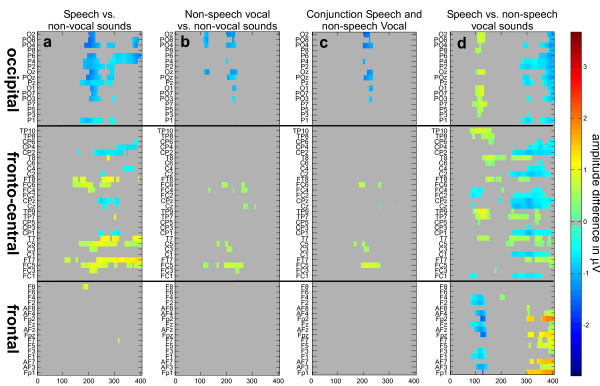
**Post-hoc results**. ERP analyses at all electrodes for the speech and non-speech vocal sounds comparisons. The color code is shown on the right side. Electrodes are stacked along the vertical axis. The horizontal black lines separate the different groups of electrodes organised in frontal, fronto-central and occipital electrodes. Gray areas were not significant. Significant ERP differences (amplitude difference in μV averaged across subjects) are represented in an increasing gradient from blue to red (jet64). Bootstrap analyses revealed significant amplitude differences between (a) speech sounds and the two non-voice categories grouped together in a conjunction test, (b) non-speech vocal sounds and the two non-voice categories grouped together in a conjunction test, c) the conjunction of a) and b), and d) the speech vs. non-speech vocal sounds test.

In order to evaluate the contribution of speech information to the voice-preferential response observed in the time-window of the auditory P2, voice stimuli were separated in speech vocal sounds (clear presence of articulated speech, n = 50) and non-speech vocal sounds (coughs, laughs, etc., n = 50). Similar patterns of amplitude difference were observed for speech and non-speech voice stimuli when compared to other categories (figure 4a and 4b). Both showed a significant difference around the P2 component starting at 164 ms (figure 4c) although effects were broader and longer lasting for speech than non-speech sounds. Finally, as one can expect, speech vs. non-speech stimuli showed significant differences over many electrodes (figure 4d). Importantly, effects appeared first between 80 and 120 ms and later on from 224 ms over fronto-temporal electrodes and from 272 ms over occipital electrodes (reversed polarity). These differences between speech and non-speech stimuli were clearly different from those observed for voices (speech and/or non-speech stimuli) compared to environmental and bird sounds.

#### Other categorical effects

In addition to the stronger voice responses reported above, another categorical effect was observed: bird songs elicited smaller N1 (100 ms) and P2 (200 ms) components than voices and environmental sounds at the vertex electrode Cz (figure 3e).

## Discussion

Scalp recordings were measured in 32 healthy adult participants to investigate the time-course of brain activity associated with the presentation of 3 categories of brief (200 ms) sounds - voices, bird songs and environmental sounds - while participants performed a pure tone detection task. We observed significantly larger ERP amplitudes for voices compared to other sound categories at fronto-temporal (positivity) and occipital (negativity) electrodes, emerging as early as 164 ms after stimulus onset and peaking around 200 ms (see figures 2, 3), an electrophysiological response termed the "fronto-temporal positivity to voices" (FTPV; [35,36]).

### Lack of voice sensitive response at electrode Cz

Results from electrode Cz did not show a consistent preference for voice over other sound categories at any latency. The bootstrap results indicated that ERPs to voices at Cz were never simultaneously larger than both bird songs and environmental sounds (figure 3e). On the contrary, bird songs elicited smaller amplitudes than both environmental sounds and voices on the N1 (~100 ms) and P2 (~200 ms) components. This effect is in line with (i) the difference in RTs showing that targets following bird songs were processed slower than when following voices or environmental sounds; (ii) subjects might have been less familiar with bird songs than with voices or environmental sounds, in line with recent findings showing an enhancement by familiarity of the N1 and the P2 components, which is predictive of the effects we observed, considering that subjects were more familiar to voices and environmental sounds [37]; (iii) acoustic analyses showing that bird songs were the most distinctive category (figure 1).

In our study, the absence of the "VSR" reported by Levy et al. (2001) around 320 ms after sound onset, could be explained by differences in materials, or experimental design, or both. In order to recognise the target and perform the task as fast as they could, subjects had to maintain their attention on every stimulus that was presented to them. This is consistent with Levy et al., (2003) who mentions that when participants attended to the stimulation sequence focusing on a feature other than timbre, the "VSR" was absent. In our experiment, because the target was a 1000 Hz pure tone, participants might have focused on pitch features, thus explaining the differences found for bird songs on the N1 and P2 (figure 3e) and the absence of a "VSR" in the latencies suggested by Levy et al., (2001). Another potential aspect leading to the differences in the scalp localisation of the voice related effects we observed and the results reported by Levy et al., (2001, 2003) is the choice of a common reference to the nose, whereas we opted for an off-line average reference [38]. Finally, the difference in findings between the studies by Levy et al. (2001, 2003) and the present study may lie in their choice of a target tone (piano tone) that belongs to the category of musical instruments, like all their stimuli except for the voices, whereas we were careful in the present study to choose a target (pure tone) that clearly did not belong to any of the three compared stimulus categories.

### Early categorical differences

The earliest categorical difference was observed at the N1 latency (starting as early as 80 ms), with smaller magnitude in response to bird songs at central and some occipital electrodes (figure 3e). These early categorical differences are comparable to latencies reported by Murray et al., (2006), who found a categorical difference between man-made and living auditory objects as early as 76 ms post sound onset.

We interpret the early birdsong ERP difference as reflecting acoustical differences between sound categories: whereas acoustic energy was concentrated at low frequencies for both voices and environmental sounds (figure 1a), it peaked at much higher frequencies for birdsongs. This finding is consistent with the well-established sensitivity of the auditory N1 component to acoustical structure [32,33]. Another early categorical difference was observed emerging 132 ms after sound onset on fronto-central electrode FC6, with a smaller negative potential in response to voices (figure 3a). This difference on the N1 component at fronto-temporal electrode FC6 could also be related to acoustical differences: lower power was observed for voices at sound onset (figure 1c). This is consistent with findings that relate the acoustic energy at stimulus onset with ERP amplitude on the N1 components [39-41].

### The FTPV: a rapid brain discrimination of sounds of voice emerging at 164 ms

The larger amplitude observed at fronto-temporal electrodes FC5 and FC6 in response to voice stimuli is consistent with our hypothesis of an early time-course for voice discrimination. As early as 164 ms post stimulus-onset, ERPs at electrodes FC5 and FC6 were consistently larger for voices than bird songs and environmental sounds. The same pattern was observed at similar latencies at occipital electrodes PO7 and PO8, with reverse polarities. Figure 3 shows an early ERP to voice at electrode locations FC5, FC6, PO8 and PO7, but this effect was also observed at the vast majority of occipital electrodes and at some frontal electrodes (figure 2c). At 200 ms, this electrophysiological response to voices reached nearly twice the amplitude of ERPs to other sounds, especially at fronto-temporal electrodes FC5 and FC6. This effect does not relate to acoustical differences, which were observed at Cz on the N1 and P2 components and at FC6 on the N1. In addition, the ERP voice response does not require subjects to make explicit discrimination among sound categories (see Methods).

The latency of this electrophysiological marker of human voice processing is in keeping with previous studies addressing complementary questions. For example, Beauchemin et al. (2006), found EEG sensitivity to voice familiarity in the time-range of the auditory P2. Although they used different stimuli, different experimental design (an oddball paradigm eliciting a Mismatch Negativity), and different EEG recording procedure (linked mastoids vs. average reference) the latencies they report are consistent with the voice effects we observed in the latencies of the auditory P2. Therefore, neuronal activity in the time window of the auditory P2 seems to be sensitive to voice vs. non-voice differences, and also higher level cognitive processes such as voice familiarity, voice identity, and voice gender [20-25].

### Voice-related brain mechanisms

This early FTPV probably corresponds to activity originating from the "what" part of the auditory stream [3-6], and most likely in the temporal voice areas [7-9]. These brain regions have been reported to be very close (two or three synapses) to core auditory regions [42] and could potentially include areas that contain a large amount of voice-selective cells as it has been demonstrated for face selective neuronal patches in the macaque brain [43], although this is very speculative and remains to be verified.

### An auditory counterpart of the face-preferential N170?

The well established face-preferential N170 ERP is characterised by larger amplitude in response to faces compared to other visual object categories from ~130 ms to 200 ms and peaking at around 170 ms after stimulus onset [44-46]. The time course of the present FTPV shows some temporal coincidence with that of the N170: significant voice/non-voice amplitude differences emerged at 164 ms post onset and were well present at several electrodes at 170 ms. Although onsets are delayed by about 30 ms, this time-course similarity between face-preferential and voice-preferential responses offers interesting avenues for future studies. Because the same broad types of information -speech, identity, affect- are typically integrated across face and voice in social interactions, a parsimonious principle of organisation would be that unimodal preferential effects for faces and voices emerge at a comparable time-frame, well-suited for integrative mechanisms [47,48]. Thus, we suggest that the FTPV could provide an auditory analogue of the well known N170 [44,49].

### The role of speech sounds

As illustrated on figure 4, speech sounds contained in the voice category contributed strongly to the FTPV. However, it also appears that vocalisations (non-speech) elicited a preferential response, although the pattern of activation was restricted to a few electrodes (in fact observed on electrodes showing the strongest averaged effect). Both observations are consistent with previous fMRI results that have shown i) a greater activity throughout the auditory cortex to speech sounds compared to their scrambled versions and ii) a greater activity to non-speech vocal sounds compared to their scrambled version restricted to the right anterior superior temporal gyrus/sulcus [50]. Indeed, our results extend fMRI ones, showing i) that the voice preferential response is not speech dependant; ii) that the preferential response for speech and non-speech stimuli has a similar time course; and iii) that the preferential response evoked for non-speech vocal stimuli compared to other sound categories is bilateral and more localised. Further experiment are needed to test if the effects observed specifically for non-speech stimuli, here for electrodes PO3/FC3-FC5, PO4/FC4-FC6, Oz-POz, correspond to activations of the anterior superior temporal gyri/sulci [50].

### Limitations

Although this study highlights for the first time an early electrophysiological response to voices, the degree of selectivity of the FTPV remains to be established. To demonstrate the robustness of the preferential electrophysiological responses in the face perception domain, several experiments were designed in order to account for the variety of visual objects [45,51-53] and uncontrolled low-level differences [46,54-58]. Future studies on voice categorisation should use a greater number of sound categories in order to better assess the robustness of this potentially selective response. Because natural sound categories are necessarily characterised by acoustical differences that may contribute to ERP differences, sound categories consisting of acoustical controls such as scrambled versions [50], or sinusoidally amplitude-modulated noise [59,60] could be used in order to rule out the contribution of factors such as amplitude modulation on the ERP.

Another way to better understand the early voice discrimination process would be to design an experiment with two stimulus categories (e.g. voice and monkey vocalisations) and at least two tasks (e.g. (i) human vs. monkey discrimination and (ii) expressions vs. no-expression discrimination) and a baseline condition, which would allow us to define whether the effect we report is specific, selective or preferential to voices, as described in [61].

Finally, an interesting possibility that remains to be tested is whether the FTPV is driven by increased attention to voices. As Levy et al. (2003) did in their study it would be interesting to manipulate attention in order to test its effect on the rapid brain discrimination of sounds of voice.

## Conclusion

We searched for early ERP markers of voice. Our results provide the first evidence of an early electrophysiological response to sounds of human voices termed the "fronto-temporal positivity to voices" (FTPV). This rapid brain response to voices appears in the latency range of the auditory P2, which is comparable to the well-known face preferential N170.

## Authors' contributions

IC, SFB, JPC, and PB designed the study. IC, SFB and JPC collected the data. IC conducted the analyses and wrote the manuscript. GAR, CRP, ML and IQ helped analyse the data. CRP, GAR, ML and PB helped revise the manuscript. All authors read and approved the final manuscript.
